# The impact of type 2 diabetes mellitus on the clinical profile, myocardial fibrosis, and prognosis in non-ischemic dilated cardiomyopathy: a prospective cohort study

**DOI:** 10.1186/s12933-024-02134-0

**Published:** 2024-02-01

**Authors:** Yangjie Li, Hong Xian, Yuanwei Xu, Weihao Li, Jiajun Guo, Ke Wan, Jie Wang, Ziqian Xu, Qing Zhang, Yuchi Han, Jiayu Sun, Yucheng Chen

**Affiliations:** 1 Department of Cardiology, West China Hospital, Sichuan University, Chengdu, 610041 Sichuan P. R. China; 2 Center of Gerontology and Geriatrics, West China Hospital, Sichuan University, Chengdu, 610041 Sichuan China; 3 Wexner Medical Center, College of Medicine, The Ohio State University, Columbus, OH 43210 USA; 4 Department of Radiology, West China Hospital, Sichuan University, Chengdu, 610041 Sichuan China

**Keywords:** Dilated cardiomyopathy, Type 2 diabetes mellitus, Myocardial fibrosis, Prognosis

## Abstract

**Background:**

The impact of the coexistence of type 2 diabetes mellitus (T2DM) in patients with non-ischemic dilated cardiomyopathy (DCM) on clinical profiles, myocardial fibrosis, and outcomes remain incompletely understood.

**Method:**

A total of 1152 patients diagnosed with non-ischemic DCM were prospectively enrolled from June 2012 to October 2021 and categorized into T2DM and non-T2DM groups. Clinical characteristics, cardiac function, and myocardial fibrosis evaluated by CMR were compared between the two groups. The primary endpoint included both all-cause mortality and heart transplantation. Cause of mortality was classified into heart failure death, sudden cardiac death, and non-cardiac death. Cox regression analysis and Kaplan-Meier analysis were performed to identify the association between T2DM and clinical outcomes. Propensity score matching (PSM) cohort including 438 patients was analyzed to reduce the bias from confounding covariates.

**Results:**

Among the 1152 included DCM patients, 155 (13%) patients had T2DM. Patients with T2DM were older (55 ± 12 vs. 47 ± 14 years, *P* < 0.001), had higher New York Heart Association (NYHA) functional class (*P* = 0.003), higher prevalence of hypertension (37% vs. 21%, *P* < 0.001), atrial fibrillation (31% vs. 16%, *P* < 0.001), lower left ventricular (LV) ejection fraction (EF) (23 ± 9% vs. 27 ± 12%, *P* < 0.001), higher late gadolinium enhancement (LGE) presence (55% vs. 45%, *P* = 0.02), and significantly elevated native T1 (1323 ± 81ms vs. 1305 ± 73ms, *P* = 0.01) and extracellular volume fraction (ECV) (32.7 ± 6.3% vs. 31.3 ± 5.9%, *P* = 0.01) values. After a median follow-up of 38 months (interquartile range: 20–57 months), 239 patients reached primary endpoint. Kaplan-Meier analysis showed that patients with T2DM had worse clinical outcomes compared with those without T2DM in the overall cohort (annual events rate: 10.2% vs. 5.7%, *P* < 0.001). T2DM was independently associated with an increased risk of primary endpoint in the overall (Hazard ratio [HR]: 1.61, 95% CI: 1.13–2.33, *P* = 0.01) and PSM (HR: 1.54, 95% CI: 1.05–2.24, *P* = 0.02) cohorts. Furthermore, T2DM was associated with a higher risk of heart failure death (*P* = 0.006) and non-cardiac death (*P* = 0.02), but not sudden cardiac death (*P* = 0.16).

**Conclusions:**

Patients with T2DM represented a more severe clinical profile and experienced more adverse outcomes compared to those without T2DM in a large DCM cohort.

**Trial registration:**

Trial registration number: ChiCTR1800017058; URL: https://www.clinicaltrials.gov.

## Background

Dilated cardiomyopathy (DCM), characterized by left ventricular (LV) enlargement and impaired contractile function unexplained by secondary causes, is one of the leading causes of heart failure (HF) [1]. The development of DCM involves a complex interplay of genetic predisposition and environmental factors [2]. Thus, DCM patients usually present with heterogeneous symptoms and clinical outcomes, which poses challenge in diagnosis and risk assessment. Notably, the coexistence of type 2 diabetes mellitus (T2DM) and HF is common, with diabetes prevalence ranging from 10% to 30% in HF cohorts [3]. Hyperglycemic status triggers a series of maladaptive stimuli that contribute to myocardial fibrosis, collagen deposition, and systolic and diastolic dysfunction, described as diabetic cardiomyopathy [4, 5]. Therefore, as a result of the combined effect of DCM and T2DM on myocardial tissue and functional remodeling, DCM patients with T2DM may suffer from worse cardiac conditions and clinical outcomes in comparison with DCM patients without T2DM.

Studies reported that diabetes was associated with symptom severity, adverse cardiac remodeling, and poor outcomes in coronary artery disease [6], hypertrophic cardiomyopathy [7], and heart failure with preserved ejection fraction (HFpEF) [8]. However, the impact of T2DM on the clinical status, myocardial tissue characteristics, and outcomes in patients with non-ischemic DCM remains incompletely understood. In this study, we aimed to provide a comprehensive evaluation of the clinical profile and prognosis in DCM patients with T2DM.

## Methods

### Study population

This study was a part of DEep phenotypic Multimodality-based ANalysis of Dilated cardiomyopathy (DEMAND) trial (Trial registration number: ChiCTR1800017058). Patients with non-ischemic DCM undergoing cardiovascular magnetic resonance (CMR) were prospectively and consecutively enrolled between June 2012 to October 2021 at West China Hospital of Sichuan University. The diagnosis of non-ischemic DCM was based on LV ejection fraction (EF) < 50% and LV diastolic dimension ≥ 55 mm without the presence of coronary disease or abnormal loading conditions. The exclusion criteria included: ischemic heart disease (defined as history of myocardial infarction or coronary artery revascularization, more than 50% luminal stenosis in any of the three main coronary arteries on coronary angiography or computed tomography, or an ischemic pattern of late gadolinium enhancement (LGE) on CMR); myocarditis; congenital heart disease; hypertrophic cardiomyopathy; cardiac amyloidosis; hypertensive cardiomyopathy; arrhythmogenic right ventricular cardiomyopathy; primary valve disease; cardiac sarcoidosis; inflammatory diseases; and estimated glomerular filtration rate < 30 ml/min/1.73m^2^. Patients with inadequate image quality, loss to follow-up, and an unwillingness to sign the informed consent were also excluded from the study. T2DM was diagnosed by the patients’ medical history, or fasting plasma glucose ≥ 7.0 mmol/L, or 2-h plasma glucose ≥ 11.1 mmol/L, or hemoglobin A1c (HbA1c) ≥ 6.5% [9]. This study complied with the Declaration of Helsinki and was approved by the institutional ethics committee of West China Hospital of Sichuan University. All enrolled patients signed the informed consent.

### CMR acquisition and analysis

CMR examination was performed on the 3.0T scanner (MAGNETOM Trio or Skyra, Siemens Healthcare, Erlangen, Germany) using a 30 or 32-channel phased array cardiac coil. Steady-state free precession (SSFP) cine images were acquired covering the LV continuously from the base to apex on short-axis views and three long-axis views (2-, 3-, and 4- chambers). LGE images were acquired using phase-sensitive inversion recovery sequence 10–15 min after gadolinium-based contrast administration. T1 mapping images were acquired at mid-ventricular short-axis slice using the modified Look-Locker inversion-recovery sequence (MOLLI). Detailed information and typical parameters were described in the previous study [10]. Ventricular volume, mass, and EF were analyzed according to the standard protocol of the Society of Cardiovascular Magnetic Resonance using Qmass (version 8.1, Medis, Leiden, Netherlands) [11]. Ventricular mass and volume were indexed to the body surface area. The presence of LGE was evaluated by two independent observers blinded to the clinical data. Extracellular volume fraction (ECV) was calculated as: ECV = (1 - hematocrit) × ([1/T1 myocardium post-contrast] - [1/T1 myocardium pre-contrast])/([1/T1 blood post-contrast] - [1/T1 blood pre-contrast]).

### Follow-up and outcomes

Patient follow-up was conducted through medical record review, consultation with attending physicians, and telephone interviews at 12-month intervals until November 2022. The follow-up was conducted by two experienced cardiologists. The composite endpoints included all-cause mortality and heart transplantation. For patients who experienced cardiovascular death, the cause of death was carefully analyzed and classified as heart failure death or sudden cardiac death (SCD) for further analysis.

### Statistical analysis

Continuous variables were displayed as means ± standard deviation or medians and interquartile range, while categorical variables were presented as numbers and percentages. Parameters were compared between groups using the Student’s *t* test or Mann-Whitney *U* test for continuous data and Chi-square test or Fisher’s exact test for categorical data, as appropriate. Imaging parameters were further compared according to diabetes status after adjusting for potential confounders using multivariate linear regression analysis. The association between variables and outcomes was estimated by using the Cox proportional hazard regression analysis. First, univariate Cox analysis was performed to find the important predictors of the composite endpoints. Then, we incorporated variables with *P* < 0.05 in the univariate analysis into the multivariable Cox regression analysis. Hazard ratio (HR) and 95% confidence intervals (CI) were reported. We also calculated the variance inflation factor to avoid collinearity. The survival curves were generated using the Kaplan-Meier analysis and compared by the log-rank test. The propensity score matching (PSM) method was conducted to control for potential confounders related to baseline characteristics of two groups, including sex, age, systolic blood pressure (SBP), New York Heart Association (NYHA) class, hypertension, atrial fibrillation, left bundle branch block (LBBB), smoking status, alcohol status, body mass index (BMI), angiotensin-converting enzyme inhibitor (ACEI)/angiotensin receptor blocker (ARB)/angiotensin receptor neprilysin inhibitor (ARNI), β-blockers, mineralocorticoid receptor antagonist (MRA), diuretics, left ventricular ejection fraction (LVEF), and LGE. We assessed the PSM in a 1:2 ratio using the nearest neighbor algorithm with a caliper width of 0.1. A two-sided *P* value of 0.05 was considered statistically significant. The statistical analyses were performed using SPSS (version 26, IBM, Chicago, USA) and R (version 4.1.1, The R Foundation for Statistical Computing, Vienna, Austria).

## Results

### T2DM prevalence and baseline characteristics

We screened 1230 patients in this prospective cohort enrolled from June 2012 to October 2021. After excluding patients with ischemic heart disease (*n* = 11), valvular heart disease (*n* = 14), hypertensive cardiomyopathy (*n* = 4), myocarditis (*n* = 3), inadequate image quality (*n* = 15), and those lost to follow-up (*n* = 31), the final cohort included 1152 patients. The mean age was 48 years, and 786 (68%) of the patients were men. Among the enrolled patients, 155 patients (13%) had coexisting T2DM.

**Fig. 1:**
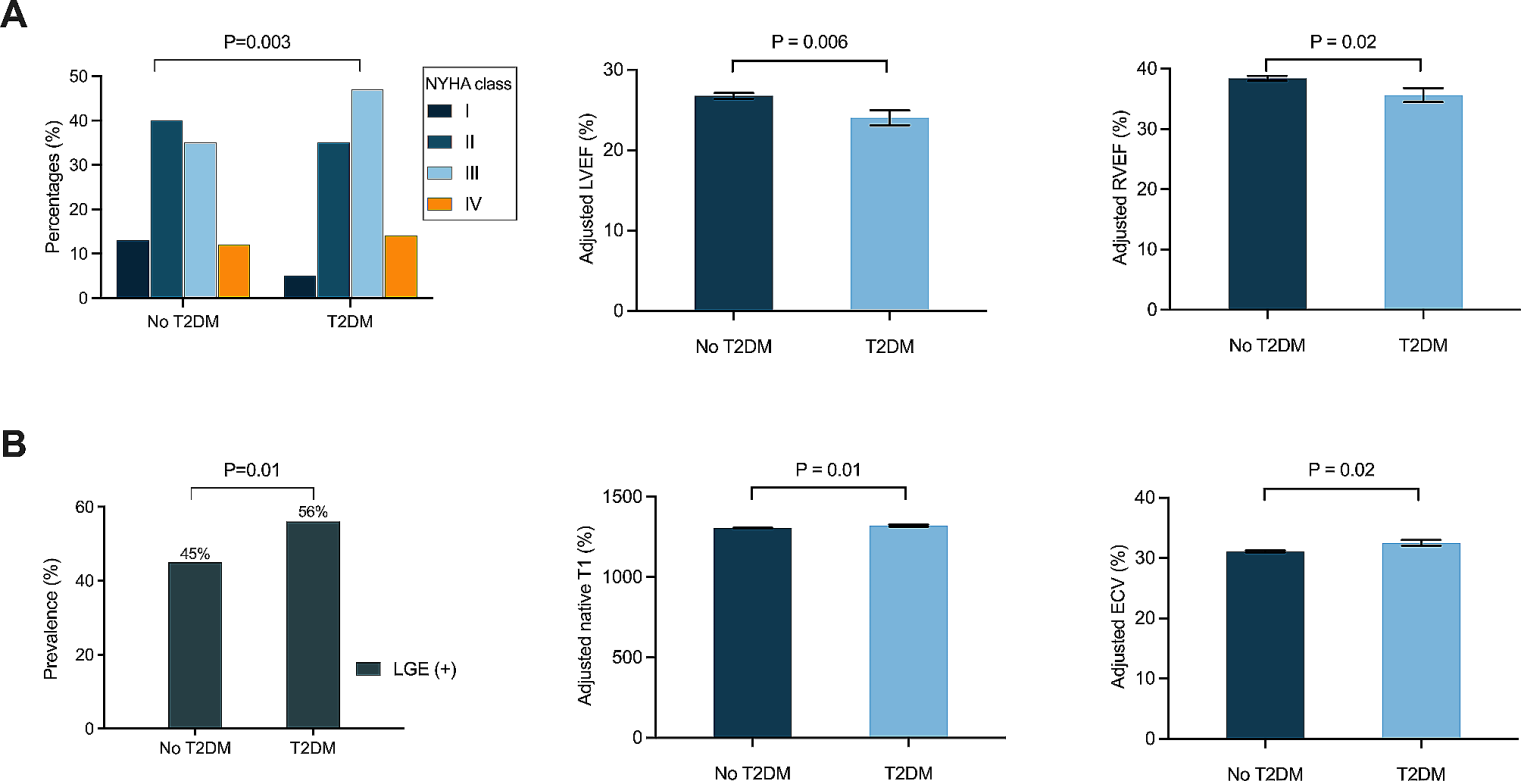
The comparison of symptom severity, cardiac function, and myocardial tissue fibrosis between type 2 diabetes mellitus (T2DM) and non-T2DM in patients with dilated cardiomyopathy (DCM). DCM patients with T2DM had worse New York Heart Association (NYHA) class, lower biventricular function (**A**), and higher degree of myocardial fibrosis (**B**). LVEF, RVEF, native T1, and ECV values were adjusted for age, sex, body mass index, systolic blood pressure, atrial fibrillation, hypertension, and heart failure drugs. LVEF, left ventricular ejection fraction; RVEF, right ventricular ejection fraction; LGE, late gadolinium enhancement; ECV, extracellular volume fraction

The baseline clinical characteristics of the entire cohort and two groups were shown in Table 1. Patients with T2DM were significantly older (mean age 55 ± 12 vs. 47 ± 14 years, *P* < 0.001) and had higher NYHA functional class (*P* = 0.003). They also had a higher prevalence of hypertension (37% vs. 21%, *P* < 0.001), atrial fibrillation (31% vs. 16%, *P* < 0.001), and were more likely to smoke (48% vs. 40%, *P* = 0.05) compared to patients without T2DM. Additionally, patients with T2DM were more frequently treated with diuretics (76% vs. 67%, *P* = 0.02), digoxin (29% vs. 21%, *P* = 0.02), and anticoagulants (27% vs. 12%, *P* < 0.001). Among patients with T2DM, 29 (19%) patients received insulin therapy. The baseline CMR characteristics of the two groups were presented in Table 2; Fig. 1. Patients with T2DM had significantly lower LVEF (23 ± 10% vs. 27 ± 12%, *P* < 0.001), right ventricular ejection fraction (RVEF) (36 ± 14% vs. 38 ± 15%, *P* = 0.05), and higher prevalence of LGE (56% vs. 45%, *P* = 0.01). They also had higher native T1 (1323 ± 81ms vs. 1305 ± 73ms, *P* = 0.01), and ECV (32.7 ± 6.3% vs. 31.3 ± 5.9%, *P* = 0.01) values. After adjustment of sex, age, BMI, SBP, atrial fibrillation, hypertension, ACEI/ARB/ARNI, β-blockers, and MRA, patients with T2DM still showed significantly lower biventricular function and higher native T1 and ECV (all *P* < 0.05). In the PSM cohort, no significant differences were observed in baseline clinical and CMR parameters between the two groups (Table 3).

**Table 1:** Baseline clinical characteristics of DCM patients with and without T2DM

	All patients	With T2DM	No T2DM	*P* value
	(*n* = 1152)	(*n* = 155)	(*n* = 997)	
Male, n	786 (68%)	115 (74%)	671 (67%)	0.09
Age (years)	48 ± 14	55 ± 12	47 ± 14	< 0.001
BMI (kg/m^2^)	24.0 ± 4.1	24.5 ± 3.9	23.9 ± 4.1	0.07
SBP (mmHg)	116 ± 17	116 ± 19	115 ± 17	0.73
DBP (mmHg)	76 ± 13	76 ± 14	76 ± 13	0.88
NYHA class, n				0.003
I	137 (12%)	7 (5%)	130 (13%)	
II	451 (39%)	55 (36%)	396 (40%)	
III	420 (37%)	72 (47%)	348 (35%)	
IV	144 (13%)	21 (14%)	123 (12%)	
Hypertension, n	266 (23%)	57 (37%)	209 (21%)	< 0.001
Atrial fibrillation, n	207 (18%)	48 (31%)	159 (16%)	< 0.001
LBBB, n	130 (11%)	17 (11%)	113 (11%)	0.89
Smoking, n	473 (41%)	75 (48%)	398 (40%)	0.05
Alcohol, n	318 (28%)	49 (32%)	269 (27%)	0.23
ACEI/ARB/ARNI, n	943 (82%)	133 (86%)	810 (81%)	0.17
β-blockers, n	950 (83%)	131 (85%)	819 (82%)	0.47
MRA, n	841 (73%)	121 (78%)	720 (72%)	0.13
Diuretics, n	785 (68%)	118 (76%)	667 (67%)	0.02
Digoxin, n	250 (22%)	45 (29%)	205 (21%)	0.02
Anticoagulants, n	164 (14%)	42 (27%)	122 (12%)	< 0.001
SGLT2i, n	89 (8%)	37 (24%)	52 (5%)	< 0.001
Biguanides, n	54 (5%)	54 (35%)	0	-
a-Glucosidase inhibitor, n	37 (3%)	37 (24%)	0	-
Insulin, n	29 (3%)	29 (19%)	0	-

Abbreviations: DCM, dilated cardiomyopathy; T2DM, type 2 diabetes mellitus; BMI, body mass index; SBP, systolic blood pressure; DBP, diastolic blood pressure; NYHA class, New York Heart Association class; LBBB, left bundle branch block; ACEI, angiotensin-converting enzyme inhibitor; ARB, angiotensin receptor blocker; ARNI, angiotensin receptor neprilysin inhibitor; MRA, mineralocorticoid receptor antagonist; SGLT2i, sodium glucose co-transporter 2 inhibitors

**Table 2:** Baseline CMR characteristics of DCM patients with and without T2DM

	All patients	With T2DM	No T2DM	*P* value
	(*n* = 1152)	(*n* = 155)	(*n* = 997)	
LVEDV (ml)	289 ± 100	297 ± 109	288 ± 98	0.35
LVESV (ml)	220 ± 99	233 ± 103	218 ± 98	0.08
LVEDV index (ml/m^2^)	171 ± 58	175 ± 63	171 ± 57	0.38
LVESV index (ml/m^2^)	130 ± 58	139 ± 60	129 ± 57	0.08
LVM (g)	143 ± 52	149 ± 50	142 ± 52	0.16
LVM index (g/m^2^)	84 ± 27	87 ± 28	84 ± 27	0.18
LVEF (%)	26 ± 12	23 ± 10	27 ± 12	< 0.001
RVEDV index (ml/m^2^)	99 ± 39	97 ± 37	100 ± 39	0.44
RVESV index (ml/m^2^)	65 ± 38	65 ± 35	65 ± 38	0.83
RVEF (%)	38 ± 15	36 ± 14	38 ± 15	0.05
LGE presence, n	534 (46%)	86 (56%)	448 (45%)	0.01
Native T1 (ms)	1308 ± 74	1323 ± 81	1305 ± 73	0.01
Post T1 (ms)	470 ± 71	445 ± 70	474 ± 70	< 0.001
ECV (%)	31.6 ± 6.0	32.7 ± 6.3	31.3 ± 5.9	0.01

Abbreviations: CMR, cardiovascular magnetic resonance; LVEDV, left ventricular end-diastolic volume; LVESV, left ventricular end-systolic volume; LVM, left ventricular mass; LVEF, left ventricular ejection fraction; RVEF, right ventricular ejection function; RVEDV, right ventricular end-diastolic volume; RVESV, right ventricular end-systolic volume; LGE, late gadolinium enhancement; ECV, extracellular volume fraction

**Table 3:** Baseline characteristics of PSM cohort

	With T2DM	No T2DM	*P* value
	(*n* = 149)	(*n* = 289)	
Male, n	111 (75%)	219 (76%)	0.77
Age (years)	54 ± 12	54 ± 13	0.64
BMI (kg/m^2^)	24.5 ± 3.6	24.4 ± 4.1	0.92
SBP (mmHg)	116 ± 19	116 ± 17	0.99
DBP (mmHg)	76 ± 14	77 ± 13	0.53
NYHA class, n			0.24
I	7 (5%)	21 (7%)	
II	53 (36%)	101 (35%)	
III	70 (47%)	114 (39%)	
IV	19 (13%)	53 (18%)	
Hypertension, n	53 (36%)	100 (35%)	0.84
Atrial fibrillation, n	43 (29%)	75 (26%)	0.52
LBBB, n	17 (11%)	35 (12%)	0.83
Smoking, n	73 (49%)	136 (47%)	0.70
Alcohol, n	47 (32%)	100 (35%)	0.52
ACEI/ARB/ARNI, n	127 (85%)	247 (86%)	0.95
β-blockers, n	126 (85%)	244 (84%)	0.97
MRA, n	116 (78%)	221 (77%)	0.75
Diuretics, n	112 (75%)	213 (74%)	0.74
Digoxin, n	40 (27%)	69 (24%)	0.50
LVEDV index (ml/m^2^)	176 ± 64	177 ± 58	0.89
LVESV index (ml/m^2^)	139 ± 60	138 ± 56	0.79
LVM index (g/m^2^)	88 ± 28	86 ± 27	0.58
LVEF (%)	24 ± 10	24 ± 10	0.85
RVEDV index (ml/m^2^)	97 ± 37	96 ± 34	0.85
RVESV index (ml/m^2^)	66 ± 36	64 ± 34	0.61
RVEF (%)	36 ± 14	36 ± 14	0.64
LGE presence, n	82 (55%)	163 (56%)	0.79
Native T1 (ms)	1318 ± 81	1310 ± 73	0.24
ECV (%)	32.9 ± 6.4	32.6 ± 6.2	0.67

Abbreviations: PSM, propensity score matching; other abbreviations as in Tables 1 and 2

### Clinical outcomes

**Fig. 2:**
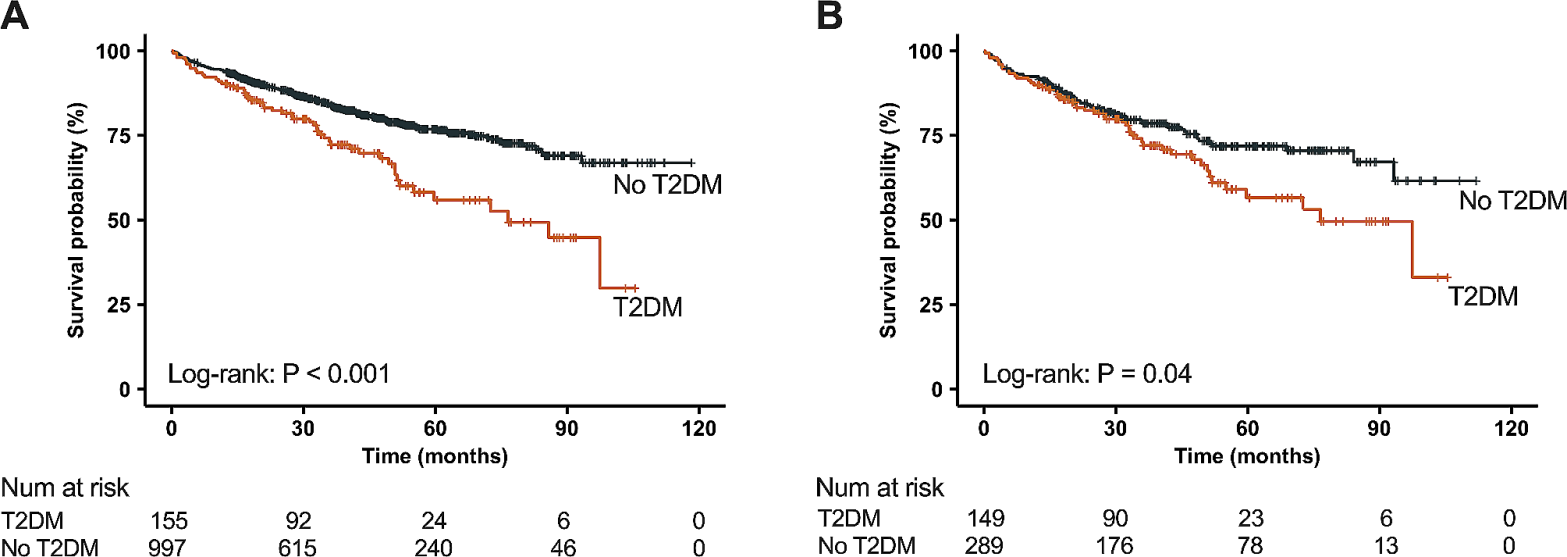
Kaplan-Meier curves of type 2 diabetes mellitus (T2DM) on composite endpoint in patients with dilated cardiomyopathy (DCM) in overall cohort (**A**) and propensity score matching (PSM) cohort (**B**)

During a median follow-up of 38 months (interquartile range: 20–57 months), the primary endpoint occurred in 239 (21%) patients. Among patients with T2DM, 50 (32%) patients reached the primary endpoint including heart failure death in 25 (16%), SCD in 13 (8%), non-cardiac death in 5 (3%), and heart transplantation in 7 (5%) patients. In patients without T2DN, 189 (19%) patients reached primary endpoint including heart failure death in 96 (10%), SCD in 60 (6%), non-cardiac death in 12 (1%), and heart transplantation in 21 (2%) patients. Kaplan-Meier curves demonstrated that patients with T2DM had a significantly higher risk of composite endpoint compared with those without T2DM in both the overall cohort (annual events rate: 10.2% vs. 5.7%, *P* < 0.001) and the PSM cohort (*P* = 0.04) (Fig. 2). Univariate Cox regression analysis showed that T2DM, age, BMI, SBP, NYHA class, hypertension, atrial fibrillation, LBBB, β-blockers, MRA, diuretics, digoxin, LV end-diastolic volume (LVEDV) index, LVEF, RVEF, LGE, and ECV were associated with the composite endpoint. In the multivariable analysis, T2DM (HR: 1.61, 95% CI: 1.13–2.33, *P* = 0.01) was an independent predictor of composite endpoint (Table 4). In PSM cohort, T2DM remained independently associated with composite endpoint (HR: 1.54, 95% CI: 1.05–2.24, *P* = 0.02) after adjustment of age, BMI, SBP, NYHA class, hypertension, atrial fibrillation, LBBB, LVEF, and LGE.

**Table 4:** Univariate and multivariable Cox regression analysis of composite endpoint

	Univariate Analysis		Multivariable analysis	
	HR (95% CI)	*P* value	HR (95% CI)	*P* value
**T2DM**	**1.89 (1.39–2.59)**	**< 0.001**	**1.61 (1.13–2.33)**	**0.01**
Male	0.95 (0.73–1.26)	0.74		
Age	1.01 (1.00–1.02)	0.03	1.00 (0.99–1.01)	0.81
BMI	0.91 (0.87–0.94)	< 0.001	0.96 (0.92–0.99)	0.03
SBP	0.97 (0.96–0.98)	< 0.001	0.99 (0.99–1.00)	0.28
NYHA class	1.87 (1.60–2.19)	< 0.001	1.09 (0.87–1.37)	0.43
Hypertension	0.62 (0.44–0.86)	0.005	1.14 (0.75–1.75)	0.54
Atrial fibrllation	2.17 (1.64–2.86)	< 0.001	1.77 (1.24–2.53)	0.003
LBBB	1.71 (1.24–2.38)	0.001	0.96 (0.63–1.45)	0.84
Smoking	1.21 (0.94–1.57)	0.14		
Alcohol	0.98 (0.73–1.29)	0.86		
ACEI/ARB/ARNI	0.74 (0.55–1.10)	0.10		
β-blockers	0.62 (0.46–0.84)	0.002	0.72 (0.50–1.06)	0.09
MRA	1.66 (1.19–2.33)	0.003	0.78 (0.51–1.19)	0.25
Diuretics	2.37 (1.67–3.35)	< 0.001	0.92 (0.57–1.47)	0.72
Digoxin	2.60 (2.01–3.37)	< 0.001	1.67 (1.20–2.33)	0.005
LVEDV index	1.01 (1.01–1.01)	< 0.001	1.01 (1.01–1.01)	< 0.001
LVEF	0.93 (0.91–0.94)	< 0.001	0.98 (0.96–1.01)	0.22
RVEF	0.96 (0.95–0.97)	< 0.001	0.99 (0.98–1.01)	0.32
LGE	2.33 (1.79–3.03)	< 0.001	1.34 (0.95–1.91)	0.10
ECV	1.11 (1.09–1.13)	< 0.001	1.07 (1.05–1.10)	< 0.001

Abbreviations as in Tables 1 and 2

**Fig. 3:**
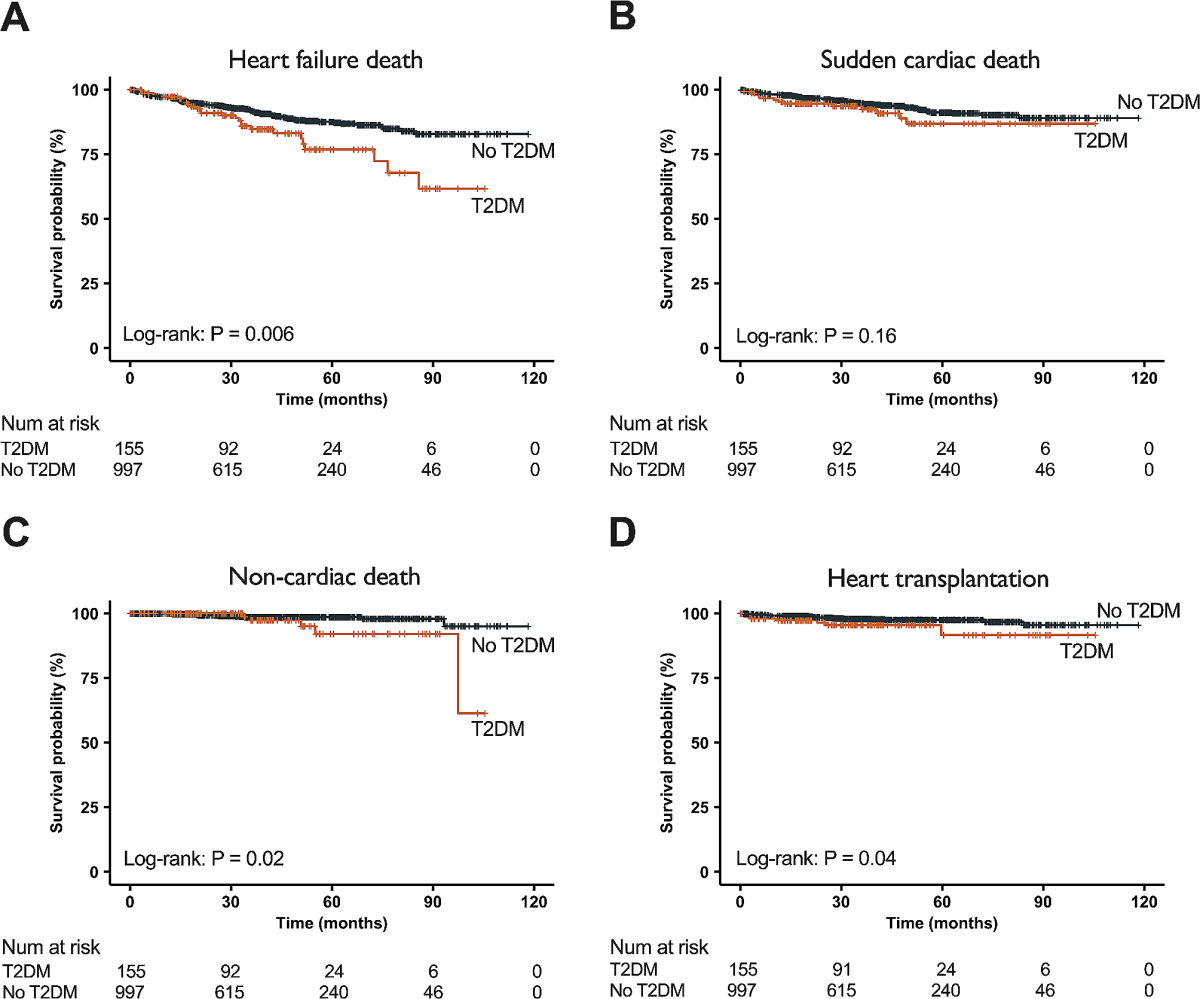
Kaplan-Meier curves for different clinical outcomes between dilated cardiomyopathy (DCM) patients with and without type 2 diabetes mellitus (T2DM). The relationship between T2DM and the risk of heart failure death (**A**), sudden cardiac death (**B**), non-cardiac death (**C**), and heart transplantation (**D**) in DCM patients

Kaplan-Meier curves revealed that patients with T2DM had a significantly higher risk of heart failure death (*P* = 0.006), non-cardiac death (*P* = 0.02), and heart transplantation (*P* = 0.04) compared to patients without T2DM. However, the two groups did not show a significant difference in the risk of sudden cardiac death (*P* = 0.16) (Fig. 3).

### Prognostic value of T2DM in subgroup analysis

**Fig. 4:**
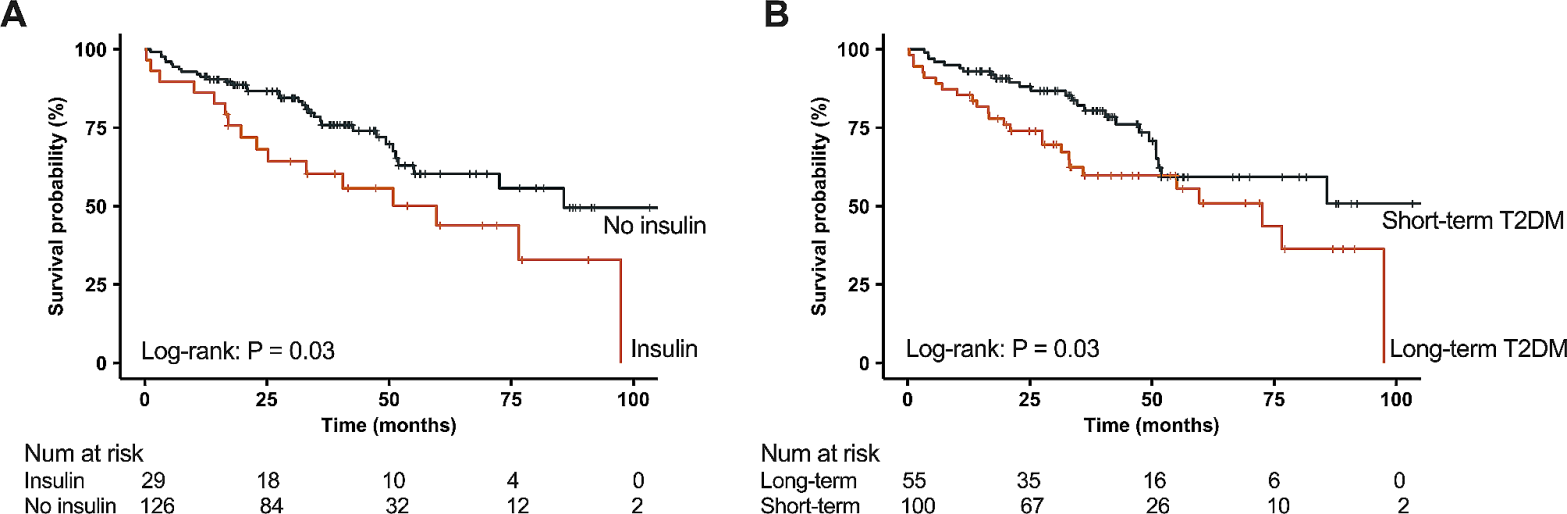
Kaplan-Meier curves of insulin use and diabetes duration on composite outcomes in dilated cardiomyopathy (DCM) patients coexisting with diabetes. Insulin usage (**A**) and long-term diabetes (diabetes duration > 4 years) (**B**) were associated with a higher risk of composite endpoint in DCM patients with diabetes

To assess the relationship between T2DM and clinical outcomes in subgroups, patients were divided into various subgroups based on sex, age, BMI, hypertension, atrial fibrillation, LBBB, LVEF, LGE, and ECV (Table 5). With the exception of females, all subgroups demonstrated an association between T2DM and higher risk of composite endpoint in patients with DCM. A significant interaction was observed between T2DM and LVEF, LGE, and ECV (all P-interaction < 0.001). T2DM was more strongly associated with the risk of composite endpoint in patients with higher LVEF (LVEF ≥ 30%) (HR 4.98, 95% CI 2.11–11.81, *P* < 0.001) compared to patients with lower LVEF (LVEF < 30%) (HR 1.45, 95% CI 1.03–2.05, *P* = 0.004). Similarly, T2DM posed a higher risk in patients with lower ECV (ECV < 30.8%) (HR 2.54, 95% CI 1.36–4.78, *P* = 0.004) than higher ECV (ECV ≥ 30.8%) (HR 1.56, 95% CI 1.06–2.32, *P* = 0.02), and in patient with negative LGE (HR 1.79, 95% CI 1.05–3.08, *P* = 0.03) than positive LGE (HR 1.76, 95% CI 1.20–2.58, *P* = 0.004). Among patients with T2DM, insulin treatment was associated with a higher risk of primary endpoint (*P* = 0.03), and patients with T2DM duration of > 4 years also showed an increased risk of clinical outcomes (*P* = 0.03) (Fig. 4).

**Table 5:** Subgroup analysis of T2DM on the composite endpoint among DCM patients

T2DM		HR (95% CI)	*P* value	*P*-interaction
Sex	Male	2.11 (1.45–3.02)	< 0.001	< 0.001
	Female	1.39 (0.71–2.71)	0.33	
Age (years)	< 60	1.78 (1.19–2.68)	0.005	< 0.001
	≥ 60	1.81 (1.10–2.98)	0.02	
BMI (kg/m^2^)	< 24	2.05 (1.39–3.04)	< 0.001	< 0.001
	≥ 24	2.02 (1.20–3.40)	0.008	
Hypertension	Yes	2.20 (1.17–4.16)	0.02	0.47
	No	2.06 (1.43–2.97)	< 0.001	
Atrial fibrillation	Yes	1.81 (1.10–2.97)	0.02	< 0.001
	No	1.61 (1.07–2.44)	0.02	
LBBB	Yes	2.74 (1.38–5.43)	0.004	< 0.001
	No	1.77 (1.24–2.51)	0.002	
LVEF (%)	< 30	1.45 (1.03–2.05)	0.04	< 0.001
	≥ 30	4.98 (2.11–11.81)	< 0.001	
LGE presence	Yes	1.76 (1.20–2.58)	0.004	< 0.001
	No	1.79 (1.05–3.08)	0.03	
ECV (%)	≥ 30.8	1.56 (1.06–2.32)	0.02	< 0.001
	< 30.8	2.54 (1.36–4.78)	0.004	

Abbreviations as in Tables 1 and 2

## Discussion

In this study, we investigated the association of type 2 diabetes mellitus with clinical profile, cardiac function, and outcomes in a large prospective dilated cardiomyopathy cohort. We found that: (1) DCM patients with T2DM exhibited worse clinical symptoms, higher prevalence of cardiovascular comorbidities, and were more often treated with diuretics and digoxin than those without T2DM; (2) DCM patients with T2DM showed impaired biventricular function and a higher degree of myocardial fibrosis compared with patients without T2DM; (3) T2DM emerged as an independent predictor of composite endpoint in DCM patients, and patients with T2DM faced a significantly higher risk of heart failure death and non-cardiac death, but not sudden cardiac death.4) In patients with lower conventional risks such as higher LVEF, negative LGE, and lower ECV, T2DM posed a stronger risk for adverse outcomes.

The intertwined relationship between heart failure and diabetes mellitus has been demonstrated in previous trials. The prevalence of HF varied between 13% and 28% in clinical trials involving patients with T2DM. On the other hand, in the general HF population, 10 − 30% patients coexisted with diabetes [3]. Moreover, HF patients were found to have a significantly higher risk of developing diabetes compared with those without HF [12]. Previous studies have highlighted the impact of T2DM on the clinical profile in HF, with patients having both conditions exhibiting more severe symptoms [3, 13]. However, studies focusing specifically on DCM patients was limited. Our results revealed that DCM patients with T2DM not only displayed worse NYHA class, but were also more likely to have hypertension and atrial fibrillation, as well as receive more intensive medical therapy. Therefore, similar to the broader HF population, DCM patients with T2DM represented a more adverse clinical phenotype. Cardiac structural and functional alterations were also more pronounced in HF patients with diabetes. Diastolic dysfunction, an early manifestation and hallmark of diabetic cardiomyopathy, included prolonged isovolumetric relaxation, altered LV filling, increased LV end-diastolic pressure, and reduced LV compliance [14, 15]. Approximately half of congestive HF patients with diabetes had diastolic dysfunction [16]. Systolic dysfunction in diabetes often takes longer to develop and occurs later than diastolic dysfunction. In our study, patients with diabetes exhibited more pronounced impairment of biventricular systolic function.

Myocardial fibrosis was an important pathophysiological feature in both diabetic cardiomyopathy and dilated cardiomyopathy. The accumulation of fibrosis and collagen contributed to the further impairment of cardiac function and poor prognosis [17, 18]. Several mechanisms may explain the cardiac fibrosis observed in diabetes, including abnormal gene expression, myofibroblast differentiation and proliferation, and recruitment of inflammation cells [4]. Li et al. found that the reduced expression of matrix metalloproteinase-2 (MMP-2) and the elevated expression of transforming growth factor (TGF)-β in diabetic conditions can induce cardiac fibrosis [19]. CMR has been considered the ideal tool for measuring myocardial tissue characteristics. LGE could be used as a surrogate for replacement fibrosis, and native T1 and ECV had a great correlation with histological collagen volume fraction and diffuse myocardial fibrosis [20]. A meta-analysis including a large number of patients (*n* = 5053) showed that diabetes had significantly higher ECV values [21]. Diabetes status and glycemic control levels were also associated with myocardial fibrosis. Patients with prediabetic status showed elevated ECV compared to the nondiabetic population [22], and HbA1c levels were positively correlated with myocardial diffuse fibrosis [23]. However, those studies mostly focused on diabetes patients without HF or decreased systolic function. Since patients with HF, especially those with DCM, already exhibited a significantly higher degree of myocardial fibrosis, whether diabetes could contribute to more severe myocardial tissue alteration remained unclear. Sakakibara et al. showed that the collagen volume fraction, as determined by histological analysis, was significantly higher in DCM patients with diabetes than patients without diabetes [24]. Our study was the first to demonstrate that patients with T2DM had significantly higher focal and diffuse myocardial fibrosis detected by CMR in a large DCM cohort.

The deleterious impact of diabetes on the clinical profile, cardiac function, and myocardial fibrosis inevitably leads to a poorer prognosis compared with patients without diabetes. Several studies have shown the association between diabetes and higher mortality risk for both heart failure with reduced and preserved EF [25–27]. However, finding regarding the etiology of HF have been conflicting. The SOLVD trial showed that diabetes increased the risk of all-cause mortality only in patients with ischemic cardiomyopathy, not in those with non-ischemic cardiomyopathy [28]. Conversely, another study demonstrated opposite results, indicating that diabetes was associated with higher mortality only in the context of non-ischemic etiology of HF [29]. Our findings showed that non-ischemic DCM patients with T2DM experienced worse clinical outcomes. In terms of the cause of death, our study revealed that diabetes increased the risk of death from pump failure and non-cardiac death, but not sudden cardiac death. This result was consistent with a previous study indicating that diabetes had no influence on ventricular repolarization and sudden cardiac death risk in a small DCM cohort [30]. Additionally, we found that T2DM had a stronger association with clinical outcomes in patients with higher LVEF, negative LGE, and lower ECV, characteristics considered otherwise low-risk with a more favorable prognosis. In addition, among patients with T2DM, insulin use and longer duration if diabetes diagnosis of greater than 4 years were associated with worse outcomes. Therefore, diabetes could serve as an additional risk stratification factor in these patients, aiding in risk prediction and clinical management.

This study had several limitations. First, this was a single-center study, and our results may not generalize to all patients. Additionally, unknown or unmeasured confounders may still have impacted analysis. Second, glycemic control level was not measured, as HbA1c was not available from all patients. Complications related to diabetes were not reported in this study. The impact of different glycemic control levels on cardiac remodeling, reversibility, and clinical outcomes should be explored in the future. Third, LGE quantification was not performed in this study based on the absence of a standardized method for LGE quantification and challenges in DCM patients due to the presence of patchy LGE regions, low scar-to-background contrast secondary to diffuse fibrosis, and thin myocardium. Instead, global ECV was measured as a surrogate for myocardial fibrosis.

## Conclusion

In patients with DCM, T2DM was associated with a more severe clinical profile, lower cardiac function, higher degree of myocardial fibrosis, and worse clinical outcomes. T2DM increases the risk of adverse outcomes in conventional low risk patients with higher EF, negative LGE, and lower ECV. Future research should explore the optimal glycemic control targets and medication effects on myocardial tissue alteration and prognosis to improve the management of patients.

## Data Availability

The datasets used and/or analyzed during the current study are available from the corresponding author on reasonable request.

## Abbreviations

DCM: Dilated cardiomyopathy
T2DM: Type 2 diabetes mellitus
CMR: Cardiovascular magnetic resonance
NYHA class: New York Heart Association class
SGLT2i: Sodium glucose co-transporter 2 inhibitors
LVEF: Left ventricular ejection fraction
RVEF: Right ventricular ejection function
LGE: Late gadolinium enhancement
ECV: Extracellular volume fraction
SCD: Sudden cardiac death
